# Insulin-like growth factor 2 as a central regulator of growth and metabolic efficiency in livestock: Genetic, nutritional, and biotechnological perspectives

**DOI:** 10.14202/vetworld.2026.1417-1436

**Published:** 2026-04-12

**Authors:** Siti Rani Ayuti, Eun Joong Kim, Sangsu Shin, Mirni Lamid, Widya Paramita Lokapirnasari, Mohammad Anam Al Arif, Sunaryo Hadi Warsito, Zulfi Nur Amrina Rosyada, Aswin Rafif Khairullah, Teuku Reza Ferasyi, Mustafa Sabri, Rimayanti Rimayanti, Wasito Wasito, Riza Zainuddin Ahmad

**Affiliations:** 1 Doctoral Program of Veterinary Science, Faculty of Veterinary Medicine, Universitas Airlangga, Jl. Dr. Ir. H. Soekarno, Kampus C Mulyorejo, Surabaya 60115, East Java, Indonesia; 2 Laboratory of Biochemistry, Faculty of Veterinary Medicine, Universitas Syiah Kuala, Jl. Tgk. Hasan Krueng Kalee No.4, Kopelma Darussalam, Banda Aceh 23111, Aceh, Indonesia; 3 Department of Animal Science and Biotechnology, Kyungpook National University, Sangju 37224, Republic of Korea; 4 Department of Animal Husbandry, Faculty of Veterinary Medicine, Universitas Airlangga, Jl. Dr. Ir. H. Soekarno, Kampus C Mulyorejo, Surabaya 60115, East Java, Indonesia; 5 Research Center for Veterinary Science, National Research and Innovation Agency (BRIN), Jl. Raya Bogor Km. 46 Cibinong, Bogor 16911, West Java, Indonesia; 6 Laboratory of Veterinary Public Health, Faculty of Veterinary Medicine, Universitas Syiah Kuala, Jl. Tgk. Hasan Krueng Kalee No.4, Kopelma Darussalam, Banda Aceh 23111, Aceh, Indonesia; 7 Laboratory of Anatomy, Faculty of Veterinary Medicine, Universitas Syiah Kuala, Jl. Tgk. Hasan Krueng Kalee No.4, Kopelma Darussalam, Banda Aceh 23111, Aceh, Indonesia; 8 Department of Veterinary Reproduction, Faculty of Veterinary Medicine, Universitas Airlangga, Jl. Dr. Ir. H. Soekarno, Kampus C Mulyorejo, Surabaya 60115, East Java, Indonesia

**Keywords:** animal growth, feed efficiency, gene polymorphism, genomic selection, *IGF2*, livestock production, muscle development, nutrient metabolism

## Abstract

Efficient livestock production depends on integrating genetic, nutritional, and management strategies to improve growth, feed efficiency, and product quality. Among genes linked to growth regulation, the insulin-like growth factor 2 (*IGF2*) gene has become a key molecular regulator affecting muscle development, nutrient metabolism, carcass composition, and overall production efficiency across various livestock species. This review summarizes current knowledge about the biological roles, genetic regulation, nutritional influences, and biotechnological uses of *IGF2* in livestock systems. *IGF2* plays a key role in prenatal and early postnatal growth by promoting myoblast proliferation, muscle fiber differentiation, and protein synthesis through major signaling pathways, including PI3K/AKT and MAPK/ERK. Genetic polymorphisms in regulatory regions of *IGF2* have consistently been linked to economically important traits such as increased body weight, improved feed conversion ratio, higher lean meat yield, and less fat deposition in cattle, pigs, poultry, and small ruminants. These findings support using *IGF2* as a molecular marker in marker-assisted and genomic selection programs aimed at boosting livestock productivity. Along with genetic factors, *IGF2* expression is heavily affected by nutritional status, environmental conditions, and epigenetic regulation. Maintaining a balanced intake of dietary energy and protein, proper management, and reducing stress levels can boost *IGF2* activity, leading to improved growth efficiency and metabolic performance. Recent advancements in biotechnology, such as genome editing, epigenetic modulation, and transcriptomic techniques, offer new ways to control *IGF2* expression for developing high-performance livestock while preserving product quality and animal welfare. However, excessive or uncontrolled modulation of *IGF2* may disrupt metabolic balance, immune function, and physiological homeostasis, highlighting the need for careful regulation and ethical consideration in genetic improvement programs. Overall, *IGF2* represents a crucial molecular link between genetics, nutrition, and physiology, and its integrated application in breeding, feeding, and biotechnology strategies offers promising prospects for sustainable and efficient livestock production.

## INTRODUCTION

Livestock growth is a fundamental aspect of the livestock industry and plays a major role in determining production efficiency and economic profitability [1]. Growth-related traits, including body weight, feed intake, and body conformation, are closely associated with production performance, particularly in meat-producing livestock species [2]. Improvement of these traits not only increases the economic value of livestock but also enhances feed utilization efficiency and supports sustainable production systems [3].

Genetic factors play a crucial role in determining individual variation in growth traits. Several candidate genes have been reported to be strongly associated with growth characteristics, including growth hormone (*GH*), growth hormone receptor (*GHR*), myostatin (*MSTN*), and insulin-like growth factor 2 (*IGF2*) [4, 5]. These genes regulate important biological processes such as metabolism, cell differentiation, muscle development, and feed conversion efficiency into body mass [6]. However, the expression of growth traits is not determined solely by genetic background, but is also influenced by environmental conditions, nutritional status, management practices, and the physiological health of livestock [7]. Animals with superior genetic potential may fail to achieve optimal growth performance if adequate nutrition and proper husbandry practices are not provided. Therefore, effective breeding programs require an integrated approach combining genetic improvement with optimized nutritional strategies and environmental management to maximize growth potential and production efficiency [8].

Improved livestock production and growth efficiency are often associated with the insulin-like growth factor (IGF) system through nutrition, genetic selection, or the application of growth-promoting strategies [9]. For example, increased *IGF1* levels have been shown to enhance growth rate and feed efficiency in beef cattle and poultry [10]. *IGF1* and *IGF2* play essential roles in regulating cell proliferation and differentiation, particularly in muscle, bone, and vital organ tissues. *IGF2* acts as a major mediator of *GH* activity and functions through systemic, paracrine, and autocrine mechanisms within body tissues [11]. Because of its central role in regulating growth and metabolism, *IGF2* has become an important research target for improving livestock productivity through natural and sustainable approaches [12].

The *IGF2* gene is influenced not only by genetic factors but also by environmental conditions such as nutrition, farm management, and stress [13]. Animals with superior genetic potential typically show faster growth rates and better feed efficiency, whereas inadequate nutrition or stressful conditions, including feed restriction or disease exposure, may decrease *IGF* expression and hinder growth performance [14]. Regulation of *IGF2* expression involves complex interactions among genetic variation, nutritional status, epigenetic modifications, and regulatory pathways across different livestock species [15]. Although many studies have explored the role of *IGF2* in individual species or specific conditions, a comprehensive overview combining genetic polymorphisms, nutritional regulation, epigenetic control, and biotechnological applications of *IGF2* across livestock species remains limited [16].

Therefore, this review compiles and compares current evidence on *IGF2*-mediated growth regulation, with particular focus on muscle development, feed efficiency, carcass quality, and responses to nutritional and environmental stress [17]. Identifying favorable *IGF2* promoter variants may provide valuable markers for selective breeding programs aimed at improving livestock productivity and efficiency [18].

Despite extensive research on growth-related genes in livestock, understanding of the role of *IGF2* in regulating growth performance, metabolic efficiency, and carcass quality remains incomplete. Most studies focus on individual species, specific polymorphisms, or isolated physiological functions, without offering a comprehensive framework that integrates genetic, nutritional, epigenetic, and biotechnological regulation of *IGF2*. Many reports examine the interaction between *IGF2* and other growth-related genes such as GH, GHR, and MSTN separately, while the coordinated regulation of these genes under different nutritional and environmental conditions has not been sufficiently summarized. Additionally, although *IGF2* polymorphisms have been linked to economically important traits, applying these findings in marker-assisted selection, genomic selection, and precision breeding programs remains inconsistent across livestock species.

Another limitation in the current literature is the lack of integrated evaluation of how nutritional status, stress, management practices, and epigenetic mechanisms influence *IGF2* expression and its downstream signaling pathways. Previous studies have demonstrated that feed composition, energy balance, and environmental stress can modify the activity of the insulin-like growth factor axis, but the combined effects of these factors on *IGF2*-mediated growth regulation have not been systematically reviewed. In addition, recent advances in biotechnology, including genome editing, transcriptomic analysis, and epigenetic modulation, have provided new opportunities to regulate *IGF2* expression; however, the potential benefits, limitations, and welfare implications of these approaches have not been critically synthesized in a single review. Therefore, a comprehensive evaluation that connects molecular mechanisms with practical applications in livestock production is still required.

Therefore, the present review aims to provide a comprehensive and integrative overview of the role of *IGF2* as a central regulator of growth, muscle development, nutrient metabolism, and production efficiency in livestock. This review summarizes current knowledge on the molecular functions of *IGF2*, its interaction with other growth-related genes such as GH, GHR, and MSTN, and its involvement in major signaling pathways controlling cell proliferation, differentiation, and protein synthesis. In addition, this review evaluates the influence of genetic polymorphisms, nutritional factors, environmental conditions, and epigenetic regulation on *IGF2* expression across different livestock species.

The review also aims to compile recent findings on the use of *IGF2* in marker-assisted selection, genomic selection, and modern biotechnological methods, including genome editing and epigenetic modulation, for enhancing growth performance and feed efficiency while safeguarding animal health and welfare. By combining information from molecular biology, animal nutrition, genetics, and biotechnology, this review strives to provide a solid scientific basis for using *IGF2* as a key target in sustainable and precision livestock production systems. Ultimately, this synthesis is expected to support future research and breeding strategies focused on improving productivity, product quality, and economic efficiency in modern livestock industries.

## REVIEW METHODOLOGY

### Literature search strategy

This review was conducted using a structured narrative approach to collect, evaluate, and synthesize scientific information related to the role of *IGF2* in livestock growth, metabolic regulation, and production efficiency. A comprehensive literature search was performed using major scientific databases, including PubMed, Scopus, Web of Science, ScienceDirect, and Google Scholar. The search covered publications from 2000 to 2025 to include both classical studies and recent advances in molecular genetics, animal nutrition, and biotechnology related to *IGF2*.

The search strategy utilized combinations of keywords and Boolean operators, including “*IGF2*”, “insulin-like growth factor 2”, “livestock growth”, “gene polymorphism”, “marker-assisted selection”, “genomic selection”, “muscle development”, “feed efficiency”, “nutrient metabolism”, “epigenetic regulation”, “CRISPR”, and “genome editing”. Additional searches were conducted using species-specific terms such as cattle, pigs, sheep, goats, and poultry. Reference lists from relevant articles were also examined to find additional publications not retrieved in the initial search.

### Inclusion and exclusion criteria

Studies were included if they met at least one of the following criteria:(i) investigated the biological function of *IGF2* in livestock or other food-producing animals,(ii) evaluated the association of *IGF2* polymorphisms with growth, carcass, reproductive, or metabolic traits,(iii) examined the influence of nutrition, management, or environmental factors on *IGF2* expression, or(iv) reported molecular, genomic, or biotechnological approaches targeting *IGF2*.

Both *in vivo* and *in vitro* studies, experimental reports, and review articles published in peer-reviewed journals were considered. Studies not related to animal production, lacking sufficient methodological details, duplicate reports, conference abstracts without full data, and non-English publications were excluded.

### Data extraction and classification

Relevant information from the selected articles was extracted and organized according to the objectives of the review. The collected data included study species, experimental approach, type of genetic or molecular analysis, nutritional or environmental factors evaluated, and the reported effects of *IGF2* on growth, metabolism, and production traits.

The selected studies were then categorized into thematic groups, including the role of *IGF2* in muscle development, regulation of nutrient metabolism, genetic polymorphisms and selection methods, effects on carcass and production traits, nutritional and environmental influences, and biotechnological interventions. This organization enabled systematic comparison among studies and helped identify consistent findings and conflicting results.

### Synthesis of evidence

The information gathered from the selected literature was critically analyzed and synthesized to provide a comprehensive overview of the regulatory role of *IGF2* in livestock production. Emphasis was placed on connecting molecular mechanisms with practical applications in breeding, nutrition, and biotechnology. The review also addressed the benefits, limitations, and potential risks tied to modulating *IGF2* expression, especially concerning animal health, welfare, and sustainable production.

This methodological approach helped identify current knowledge gaps and provided a scientific foundation for future research and the development of precision livestock improvement strategies targeting *IGF2*.

### *IGF2* ROLE IN MUSCLE DEVELOPMENT

Livestock growth is a critical determinant of animal production system productivity and economic efficiency. Growth traits, such as body weight and size, have a direct impact on production efficiency and farmer profits [19]. Optimal body weight and body size are related to meat production capacity and feed utilization efficiency. Improving growth-supporting factors is crucial for improving livestock quality and quantity [20]. Genetic background plays a fundamental role in shaping growth potential among these factors. Numerous studies have demonstrated that variation in specific candidate genes significantly contributes to differences in the growth rate and body development of livestock populations [21]. Genes associated with GH regulation, protein metabolism, and digestive efficiency have a significant impact on growth trait expression [22]. Consequently, identifying favorable genetic variants enables a more precise selection of superior animals for use in targeted breeding programs [23].

*IGF2* stimulates myoblast growth through the PI3K/AKT and MAPK/ERK pathways [24]. It is secreted by various cell types and interacts with IGF1R or *IGF2*R receptors, activating signaling pathways such as PI3K/AKT and MAPK/ERK [25]. The activation of these pathways promotes gene transcription involved in myogenesis, protein synthesis, and satellite cell proliferation, thereby aiding the formation and maturation of muscle fibers. *IGF2* is highly expressed and serves as a key regulator in early skeletal muscle mass development [26]. It encourages the division of muscle precursor cells (myoblasts) and their transition into myotubule cells, which eventually develop into functional muscle fibers [27]. The decrease in *IGF2* expression during the postnatal stage indicates that its role is prominent during early muscle development and lessens in postnatal muscle growth, though it still supports muscle tissue regeneration [28]. Using *IGF2* promoter variants in MAS programs remains promising. This approach could improve production efficiency and farmer welfare [29, 30]. Genomic selection and biotechnology can accelerate achieving improved breeding targets. Factors such as feed quality, health management, and environmental conditions influence the expression of growth-related genes [31].

From an economic perspective, enhancing growth traits through genetically informed selection can significantly boost farm profitability. Livestock with faster growth rates and larger body sizes generally command higher market value, while improved feed efficiency cuts production costs [32]. Choosing individuals with better feed efficiency can lower costs and increase the profitability of livestock operations. Adopting genetic selection-based breeding strategies can help boost the competitiveness of the livestock industry [33]. Additional research is needed to find the most effective genetic variants for improving livestock growth traits. Understanding genetic and epigenetic interactions under various environmental conditions is also crucial for optimizing breeding strategies [34].

Improving livestock growth efficiency through genetic marker-based breeding can deliver significant benefits to farmers. Livestock with faster growth rates can reach market weight sooner, reducing maintenance costs and boosting production efficiency [35]. Additionally, using genetic selection can lessen dependence on traditional methods that are more time-consuming and less consistent. Challenges in adopting this technology, such as the cost of genetic analysis and limited access in some regions, must be addressed for broader implementation [36]. It is crucial to tackle these issues through coordinated efforts among scientific research, policy making, and technology transfer [37]. By combining genetic-based breeding with precision management practices, the livestock industry can move toward more efficient, sustainable, and globally competitive production systems [38].

## *IGF2* IMPACT ON MUSCLE FIBER COMPOSITION

The *IGF2* gene is a key regulator of muscle growth and metabolism in cattle, pigs, sheep, and chickens. Extensive evidence demonstrates that elevated *IGF2* expression is positively associated with enhanced protein synthesis and myoblast proliferation, leading to increased muscle accretion and an improved M/F ratio [39]. Consequently, *IGF2* has been widely recognized as a major genetic determinant of carcass quality traits across multiple livestock species [40]. Mutations in the *IGF2* regulatory region can significantly increase muscle growth while reducing subcutaneous fat content [41]. For example, specific mutations in *IGF2* are associated with a 15%–20% reduction in back-fat thickness without compromising meat quality in pigs [42]. Similarly, *IGF2* gene polymorphisms in cattle have been correlated with economically important traits such as marbling, meat tenderness, and feed efficiency. These findings underscore the potential of *IGF2*-based genetic selection strategies to enhance meat production quality and quantity in livestock populations [43].

*IGF2* plays a role not only in muscle growth but also in energy metabolism and body composition regulation. The *IGF2* hormone produced by this gene exerts a strong anabolic effect, aiding in increasing the efficiency of feed conversion into muscle mass [44]. *IGF2* is a key growth factor involved in the proliferation and differentiation of muscle, bone, and internal organs [45]. It remains active in supporting hypertrophy by activating the PI3K/AKT/mTOR signaling pathway, which boosts protein synthesis and muscle growth. Additionally, *IGF2* interacts with transcription factors such as Myogenin and MyoD, which are involved in controlling muscle cell differentiation and maturation [46].

**Figure 1:**
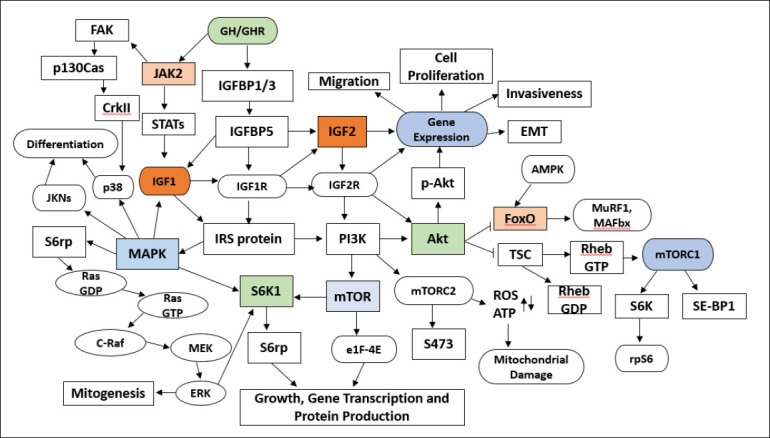
Role of insulin-like growth factor 2 in the regulation of cell signaling pathways and growth mechanisms [Source: Figure prepared by Siti Rani Ayuti].

Moreover, the *IGF2* gene plays a key role in muscle growth, cell differentiation, and energy metabolism, making it a prime candidate as a molecular marker for livestock meat production. *IGF2* increases protein synthesis and muscle cell proliferation, thereby directly influencing the meat-to-fat ratio and carcass quality [47]. Numerous quantitative genetic studies have linked *IGF2* to QTLs associated with growth performance, feed efficiency, and carcass characteristics. The identification of a quantitative trait nucleotide (QTN) within the *IGF2* regulatory element has provided critical insights into how subtle genetic variations can induce substantial changes in gene expression and phenotypic outcomes [48]. The QTN in the *IGF2* regulatory element was discovered through genome-wide association studies and QTL mapping, which aimed to link genetic variation to livestock production performance. This mutation is located in the regulatory region of *IGF2* expression, which controls gene transcription levels in various tissues [49] (Figure 1).

Mutations in this region influence gene expression by altering interactions with transcription factors and epigenetic mechanisms, including DNA methylation and histone modifications [50]. The discovery of QTN mutations in *IGF2* offers significant benefits in GS and MAS-based breeding [51]. Breeders can select individuals with favorable *IGF2* alleles using genetic data to improve growth efficiency, carcass quality, and the balance between muscle and fat mass. Cattle carrying beneficial *IGF2* mutations exhibit improved feed conversion and endurance, making them a prime target in modern breeding programs [52]. Livestock carrying advantageous *IGF2* variants consistently exhibit enhanced muscle hypertrophy, increased daily weight gain, and superior feed conversion efficiency. These animals can utilize nutrients more effectively, achieving accelerated growth rates without disproportionate increases in feed intake [53]. Collectively, these attributes position *IGF2* as a central genetic target in modern livestock breeding programs aimed at optimizing meat production and economic efficiency [54].

### *IGF2* EXPRESSION AND MEAT QUALITY IMPROVEMENT

The *IGF2* gene plays a fundamental role in ensuring proper organogenesis and tissue development, including the formation of skeletal muscle, cardiac tissue, and the liver [55]. Elevated *IGF2* expression during early developmental stages supports the regulation of body size and promotes the formation of larger muscle mass [56]. In addition, *IGF2* regulates energy balance in the chicken body by influencing glucose metabolism and body fat regulation. *IGF2*, together with other hormones such as insulin, ensures efficient energy management in the body [57]. This regulatory mechanism is crucial in chickens because *IGF2* contributes to the conversion of feed into energy used for body growth. This hormone allows chickens to optimize the use of available energy, supporting faster and more efficient growth, which increases livestock productivity [58]. The importance of *IGF2* in chicken growth and development opens up opportunities for its application in genetic-based breeding programs. Identification of *IGF2* gene variations can be used in MAS technology-assisted selection to improve chicken growth traits. In addition, genetic editing, such as CRISPR–Cas9, can be used to increase *IGF2* expression in chickens, which in turn can accelerate growth rates and improve production efficiency [59]. Thus, *IGF2* not only plays a central role in natural biological processes but also offers great potential for optimizing chicken breeding outcomes for more efficient production [60].

Polymorphisms within the *IGF2* gene, especially in promoter and regulatory regions, have been linked to differences in growth traits like birth weight and postnatal body size across various livestock species, including chickens [61]. These links further support *IGF2* as a candidate gene for marker-assisted selection in livestock improvement efforts [62]. *IGF2* activates signaling pathways that affect cell metabolism and encourage cell proliferation and differentiation in chickens. As a polypeptide hormone involved in regulating overall growth, *IGF2* ensures that cell division and growth happen efficiently and in an organized way during chicken development [63]. Besides its role in promoting cell growth, *IGF2* also plays a key part in mediating the effects of the *GH* gene [64].

Growth hormones stimulate the formation of new tissue and maintain existing tissue, particularly in increasing muscle mass and regulating metabolism [65]. *IGF2* acts as a mediator that amplifies the effects of GH in this process. In this case, *IGF2* accelerates the formation of larger muscle tissue, which in turn increases the overall body size of chickens [66]. Therefore, *IGF2* is crucial for speeding up growth, especially during the early stages of life. *IGF2* plays an essential role in regulating the formation of muscle and fat tissue during chicken development [67]. Chickens can develop greater muscle mass through increased *IGF2* expression, leading to higher body weight and a more optimal body size. High *IGF2* expression can result in increased protein levels in muscles and other tissues, supporting overall body growth [68]. *IGF2* also contributes to bone development, ensuring that the skeletal structure develops properly to support the larger body of the chicken. The *IGF2* gene not only promotes muscle growth but also helps other organs and body systems develop properly during growth [67].

The *IGF2* gene is also recognized as an important regulator of growth and development across multiple livestock species because of its imprinting characteristics [68]. This phenomenon occurs in certain genes involved in regulating body growth and development, where the parental origin of the allele influences gene expression. This opens the possibility for a deeper understanding of *IGF2* gene expression regulation in chickens and its role in growth regulation [69]. Further investigation of *IGF2* expression patterns in chickens using advanced molecular approaches, such as quantitative real-time polymerase chain reaction (PCR) and RNA sequencing, has confirmed its significant role in growth regulation [70]. Understanding the genetic and epigenetic factors that influence *IGF2* expression provides valuable insights for developing more precise and effective breeding strategies [71]. Elucidating *IGF2*-mediated regulatory mechanisms holds considerable promise for improving both the quality and quantity of poultry production through genetically informed selection and breeding practices [72].

## NUTRITIONAL REGULATION OF *IGF2* EXPRESSION

The *IGF2* gene is a crucial growth factor that plays a central role in regulating nutrient metabolism in livestock. As a peptide hormone, *IGF2* is involved not only in growth and development but also in energy metabolism and nutrient use [73]. A thorough understanding of the molecular mechanisms through which *IGF2* influences nutrient metabolism is vital for enhancing livestock productivity via integrated nutritional and genetic strategies [74]. This highlights *IGF2* as a key factor in boosting livestock growth and efficiency. Higher *IGF2* expression is linked to increased body weight and lean muscle mass. Therefore, genetic selection targeting *IGF2* could be an effective strategy to improve high-quality meat production in livestock [75].

*IGF2* regulation is also closely linked to feed efficiency and nutrient utilization. This factor enhances the use of amino acids and glucose in cells, supporting more efficient growth [76]. Manipulating *IGF2* expression through targeted nutritional strategies offers a practical way to improve feed efficiency while also decreasing nitrogen waste and reducing environmental impact. Optimizing *IGF2* activity could thus be a sustainable method to boost livestock production and lessen the environmental footprint [77]. Since *IGF2* plays a key role in managing nutrient metabolism, there is considerable potential to enhance livestock productivity through genetic selection combined with nutritional management [78]. Interactions among *IGF2* and environmental, nutritional, and genetic factors provide valuable insights into the regulation of growth and metabolic efficiency in livestock [79]. At the cellular level, *IGF2* influences metabolism via complex signaling pathways that coordinate nutrient use, improve feed conversion, and regulate protein synthesis and breakdown [80].

One of the main functions of *IGF2* in nutrient metabolism is to promote protein synthesis and reduce muscle protein breakdown. This process is essential for supporting livestock growth, especially during rearing and fattening stages [81]. *IGF2* works together with other growth hormones, such as IGF1 and insulin, to enhance amino acid uptake and encourage muscle fiber development, highlighting its potential as a genetic target for boosting meat production efficiency [82]. Besides its role in protein metabolism, *IGF2* also significantly affects energy metabolism and glucose use. It increases insulin sensitivity, which speeds up glucose absorption and storage in muscle and liver tissues [83]. This improves how efficiently energy from feed is used, directly impacting growth and overall health [84]. Higher *IGF2* levels may also decrease the need for fat tissue as an energy source, allowing more dietary energy to be used for muscle building [85].

Integration of *IGF2*-targeted genetic selection with optimized nutritional strategies offers a strong approach for improving livestock production efficiency and sustainability [86]. The *IGF2* gene plays a crucial role in regulating nutrient metabolism in livestock by acting as a key mediator in metabolic pathways influencing muscle growth, protein synthesis, and feed conversion efficiency [87]. *IGF2* stimulates cellular proliferation and differentiation through interaction with specific receptors, particularly in skeletal muscle and metabolically active organs such as the liver and pancreas. In addition to protein metabolism, *IGF2* influences energy metabolism, especially glucose and lipid utilization [88]. *IGF2* increases insulin sensitivity, allowing muscle cells to absorb and utilize glucose more efficiently, thereby reducing excessive fat accumulation and directing more energy toward muscle growth [89]. Appropriate nutritional strategies, including balanced carbohydrate and protein formulation, can maximize *IGF2* expression and improve production efficiency [90]. Overall, understanding the mechanisms by which *IGF2* regulates nutrient metabolism provides important opportunities to improve livestock productivity through scientifically based nutritional and genetic interventions [91]. Interaction between metabolic pathways and *IGF2* represents an important strategy to optimize growth, increase feed efficiency, and produce higher-quality livestock products [92].

## *IGF2* POLYMORPHISMS AND ASSOCIATED GENETIC STRATEGIES

The *IGF2* gene plays a crucial role in regulating growth, metabolism, and reproductive performance in livestock, particularly poultry. Polymorphisms in the *IGF2* gene, especially single-nucleotide polymorphisms (SNPs), have been identified as potential markers of reproductive traits in poultry [93]. Variations in regulatory regions, such as promoter and intronic regions, can influence *IGF2* expression. These genetic variations provide valuable information for marker-assisted selection (MAS), enabling breeders to selectively improve production efficiency [94]. Multiple *IGF2* polymorphisms exert substantial effects on meat production traits across livestock species. For example, in the swine industry, specific mutations in the *IGF2* regulatory region have been associated with increased muscle growth and decreased back-fat deposition, resulting in more efficient carcasses [95].

*IGF2* also affects follicular development, hormone secretion, and overall reproductive success in poultry. This gene encourages cell growth and specialization and helps maintain healthy ovarian tissue function, which is vital for egg production [96]. Recent research has found specific *IGF2* SNP linked to increased egg production, better eggshell quality, and longer reproductive lifespan in poultry [97]. These polymorphisms assist in identifying livestock with superior genetic traits, making them useful markers for selective breeding programs [98].

Mapping *IGF2* polymorphisms can be used in genome-based breeding to produce livestock with better meat qualities. *IGF2* activates the PI3K/AKT/mTOR signaling pathway by binding to the IGF1R receptor, which stimulates protein synthesis and muscle growth [99]. Additionally, *IGF2* interacts with IGF-binding proteins (IGFBPs), which regulate its bioavailability and tissue-specific activity. Functional genomics and transcriptomic methods offer powerful tools for understanding how specific SNPs affect *IGF2* expression, signaling efficiency, and reproductive physiology [100].

Polymorphisms in the *IGF2* gene influence various biological functions, especially those related to reproductive traits in poultry. Genetic variations, including SNPs, can change gene expression and function [101]. Specific *IGF2* polymorphisms have been linked to higher egg-laying rates, better eggshell quality, and a longer reproductive period [102]. Certain SNPs can boost *IGF2* expression in ovarian tissue, promoting follicular development and increasing ovulation frequency [103]. Identifying and understanding these polymorphisms enable breeders to use genetic selection to improve reproductive traits like egg size and production efficiency [104].

Although current research emphasizes the potential of *IGF2* polymorphisms to enhance egg production traits, many aspects still need exploration. Future studies should examine broader genetic and environmental interactions that influence *IGF2* expression and its role in reproductive performance [105]. Investigating the interactions between *IGF2* and genes involved in steroid hormone synthesis or yolk formation could provide deeper insights into the genetic networks controlling egg-laying efficiency [106]. The important role of *IGF2* in avian reproductive physiology further underscores its potential to improve productivity through polymorphisms [107]. *IGF2* polymorphisms are also linked to variations in body size and growth, with some allelic variants being beneficial for faster growth and better feed efficiency [108]. Therefore, *IGF2* functions not only as a regulator of growth and reproduction but also as an essential genetic factor in overall production performance [109]. Overall, these findings suggest that *IGF2* is a promising molecular target for MAS aimed at improving key traits in modern poultry breeding programs [110].

## BIOTECHNOLOGICAL INTERVENTIONS TARGETING *IGF2*

Genetic engineering is an innovative approach to increase *IGF2* expression to boost livestock growth efficiency and nutrient metabolism [111]. Because of the central role of *IGF2* in regulating muscle development, energy metabolism, and feed conversion efficiency, targeted manipulation of its expression can significantly enhance livestock productivity [112]. Modern genetic engineering methods allow precise control of *IGF2* through genome editing technologies such as CRISPR–Cas9, epigenetic regulation, and transgenic approaches [113]. In various livestock species, increasing *IGF2* expression via CRISPR–Cas9 has been shown to promote muscle growth, improving carcass quality and meat production [114].

In addition to CRISPR–Cas9 techniques, epigenetic approaches are also promising for modifying *IGF2* expression [115]. Epigenetic manipulation through nutritional input or molecular intervention can naturally increase *IGF2* expression, allowing livestock to grow faster and more efficiently without permanent genetic modification [116]. This approach is considered safer and more widely applicable in the livestock industry because regulatory restrictions are less strict than those for conventional genetic engineering technologies [117]. With advances in molecular technologies, modifying *IGF2* expression has become a strategic method for improving livestock productivity. Genetic engineering has thus become an important tool for enhancing *IGF2* activity to support growth and metabolic efficiency [118]. One major advantage of increasing *IGF2* expression through CRISPR–Cas9 is optimizing carcass composition, where more nutrients are directed toward muscle growth rather than fat deposition [119]. The meat-to-fat ratio is a key factor in determining product quality and market value in the livestock industry. Therefore, improving *IGF2* activity not only boosts production efficiency but also meets consumer demand for healthier and higher-quality products [120].

Beyond CRISPR–Cas9 and traditional transgenic methods, a wide array of molecular and genomic technologies has been used to modify *IGF2* activity in livestock species (Table 1) [121–129]. These approaches work at various regulatory levels, from gene silencing to genome editing and advanced selection tools, showcasing the versatility of *IGF2* as a biotechnology target.

**Table 1:** Applied genetic technologies to enhance *IGF2* function.

Genetic technology	Short work principle	Livestock species	*IGF2* target	Observation outcome	Reference
RNA interference	Specific reduction in *IGF2* expression	Chicken	*IGF2* mRNA	Regulation of muscle growth	[121]
CRISPR/Cas9	Direct *IGF2* gene editing	Pig	Exon 1	Increased muscle mass and feed efficiency	[122]
Marker-assisted selection	Selection-based on the *IGF2* SNP markers	Cow	SNP *IGF2*	Increased carcass weight and *IGF2* expression from both alleles (biallelic expression)	[123]
Epigenetic Modification	The methylation/demethylation locus of *IGF2*	Sheep	ICR Region	Activation of *IGF2* expression	[124]
Transgenesis	Insertion of an extra *IGF2* gene copy	Goat	*IGF2* Promoter	Faster and more uniform muscle growth	[125]
Gene Promoter Engineering (GPE)	Promoter element engineering to enhance the expression of	Cow	Promoter region	Increased *IGF2* expression	[126]
Synthetic mRNA therapy	Administration of artificial *IGF2* mRNA for transient gene expression	Chicken	*IGF2* transcript	Acceleration of the early growth phase	[127]
GWAS-Guided Selection	Livestock selection-based on the association of SNPs with *IGF2*	Pig	Intronic *IGF2* SNP	Increases *IGF2* activity and muscle growth	[128]
miRNA Modulation	Inhibition of *IGF2*-negative regulatory miRNA	Sheep	miR-675	Increases *IGF2* mRNA stability and protein translation	[129]

Technologies listed are based on published experimental or review data; outcomes represent reported effects in the cited studies. *IGF2* targets refer to the specific genetic element modified or selected (e.g., coding region, regulatory region, or transcript). Observation outcomes summarize the primary reported benefit related to muscle growth, meat production, or related traits. References correspond to the numbered list provided in the manuscript. Abbreviations: CRISPR/Cas9 = clustered regularly interspaced short palindromic repeats/CRISPR-associated protein 9, GWAS = genome-wide association study, ICR = imprinting control region, *IGF2* = insulin-like growth factor 2, miRNA = microRNA, SNP = single-nucleotide polymorphism.

refer to the specific genetic element modified or selected (e.g., coding region, regulatory region, or transcript). Observation outcomes summarize the primary reported benefit related to muscle growth, meat production, or related traits. References correspond to the numbered list provided in the manuscript. Abbreviations: CRISPR/Cas9 = clustered regularly interspaced short palindromic repeats/CRISPR-associated protein 9, GWAS = genome-wide association study, ICR = imprinting control region, *IGF2* = insulin-like growth factor 2, miRNA = microRNA, SNP = single-nucleotide polymorphism.

RNA interference (RNAi) represents one of the earliest functional genomic tools used to investigate the regulation of *IGF2*. Targeted suppression of *IGF2* mRNA through RNAi has clarified its role in muscle growth regulation and developmental signaling pathways in chickens [121]. Although RNAi mainly serves as a research tool, it provides important mechanistic insight into *IGF2*-mediated anabolic processes.

Genome editing with CRISPR/Cas9 allows precise and permanent changes to the *IGF2* gene. In pigs, editing exon-1 directly has led to increased muscle mass and better feed efficiency, stressing the economic value of *IGF2*-focused techniques in commercial swine farming [122]. Besides direct editing, MAS has been used in cattle by identifying advantageous *IGF2* SNPs, which correlate with higher body weight, improved carcass traits, and increased gene activity [123].

Epigenetic modification further broadens the possibilities for *IGF2* regulation without changing the DNA sequence. In sheep, methylation and demethylation of the imprinting control region activate *IGF2* expression, showing how epigenomic remodeling can impact muscle development and growth traits [124]. Similarly, transgenic methods involving the insertion of additional *IGF2* gene copies in goats have led to faster and more consistent muscle growth, confirming the anabolic role of sustained *IGF2* overexpression [125].

At the transcriptional level, promoter engineering has been utilized in cattle to increase *IGF2* expression [126]. Synthetic mRNA therapy has also become a temporary and reversible approach, where administering artificial *IGF2* mRNA to chicken speeds up early growth without causing permanent changes to the genome [127].

Genomic selection approaches also contribute to optimization of *IGF2*. Genome-wide association studies in pigs have identified intronic SNPs associated with increased *IGF2* activity and enhanced muscle growth [128]. Post-transcriptional regulation through microRNA modulation has also been reported, where inhibition of miR-675 improves *IGF2* mRNA stability and translation efficiency, thereby promoting muscle accretion [129].

In addition to production benefits, modulation of *IGF2* expression can also enhance livestock health and welfare [130]. Excessive fat buildup is often linked to metabolic disorders, lower endurance, and higher mortality rates [131]. Proper regulation of *IGF2* may help livestock achieve healthier body composition, better endurance, and higher-quality meat [132]. This approach can also promote more stable large-scale production, boosting the global competitiveness of the livestock industry [133]. Overall, CRISPR–Cas9, epigenetic modulation, and integrated biotechnological strategies targeting *IGF2* offer a strong framework for improving meat production efficiency without sacrificing animal health or product quality [134]. When combined with optimized nutrition and management, *IGF2*-based interventions are a promising way to achieve sustainable, competitive, and cost-effective livestock production systems [135].

## EXPRESSION OF *IGF2* AND LIVESTOCK PERFORMANCE

*IGFs*, especially *IGF1* and *IGF2*, are peptide hormones that control growth, development, and metabolism in livestock [136]. These hormones serve as key mediators of GH activity and are structurally similar to insulin. While *IGF1* is mainly produced in the liver in response to GH stimulation, *IGF2* plays a more significant role in fetal and early postnatal development, influencing long-term growth potential and metabolic programming [137]. IGFs act through specific receptors, mainly *IGF1R*, to promote cell proliferation, differentiation, and survival in various tissues. Their production and activity are affected by various physiological and environmental factors, including nutritional status, GH levels, and stress [138]. *IGF1* production is regulated by the GH–IGF axis via complex feedback mechanisms that maintain growth homeostasis. Additionally, IGFs have autocrine and paracrine actions that regulate local growth in tissues such as skeletal muscle, bone, and adipose tissue [139]. Disruption of this system can noticeably impact growth performance and metabolic efficiency in animals. The biological activity of IGFs is strongly affected by six types of binding proteins called insulin-like growth factor-binding proteins (IGFBPs) [140]. These IGFBPs refine IGF signaling and influence tissue-specific growth responses and metabolic regulation through these mechanisms [141].

Experimental evidence from both *in vivo* and *in vitro* studies further substantiates the functional importance of *IGF2* in regulating growth performance and metabolic activity in livestock species (Table 2) [142–149]. *In vivo* investigations demonstrate that modulation of *IGF2* expression directly influences productive traits and tissue development. In transgenic pigs, elevated *IGF2* expression has been associated with improved feed conversion ratio (FCR) and enhanced meat aging quality, supported by carcass evaluation, real-time quantitative polymerase chain reaction (RT-qPCR), and muscle histology findings [142]. Similarly, studies in layer chickens have shown that *IGF2* expression during early developmental stages contributes to optimal digestive tract growth, indicating its role in organogenesis and nutrient absorption capacity [143]. In dairy cows, *IGF2* expression has been positively correlated with energy balance status and milk production, where higher expression levels were observed under positive energy conditions, accompanied by improved lactational performance and metabolic indicators [144].

**Table 2:** Experimental studies on the function of *IGF2* in livestock.

Type of study	Sample	Research purposes	Results	Research methods	Reference
*In vivo*	Transgenic Pigs	Growth and meat quality evaluation	Improved aging quality and FCR	Carcass analysis, RT-qPCR, and muscle histology	[142]
	Layer chicken	Expression of *IGF2* and early organ development	Optimal growth of the digestive tract	RT-qPCR and intestinal histology analysis	[143]
	Dairy cows	*IGF2* and its relationship with energy metabolism and milk production	High *IGF2* expression when energy is positive increases milk production	RT-qPCR analysis and blood metabolites	[144]
*In vitro*	Chicken muscle cell culture	Assessing the activation of the *PI3K-AKT-mTOR* pathway	*IGF2* accelerates protein synthesis in muscles	Western blot analysis (p-AKT, p-mTOR), RT-qPCR, and immunofluorescence	[145]
	Goat liver cell culture (Figure 1)	Role of *IGF2* in lipid metabolism regulation	Decreased lipid accumulation in hepatocytes	Oil Red O staining and RT-qPCR	[146]
	Duck muscle progenitor cells	Stimulation of muscle cell differentiation by *IGF2*	Increased myotube formation	Immunofluorescence and Western blot analysis	[147]
	Bovine myoblast culture	*IGF2* cell proliferation assay	Increases muscle proliferation and protein synthesis	RT-qPCR, Western blotting, and MTT assays	[148]
	Porcine embryo fibroblast cells (Figure 1)	Assessing *IGF2* downstream gene expression in metabolic pathways	*PI3K/AKT* pathway activation and increased *GLUT4* expression	Western blot and quantitative PCR targeting *PI3K/AKT/GLUT4*	[149]

*In vivo* studies were conducted in live animals; *in vitro* studies used primary or immortalized cell cultures. Research purposes indicate the main objective(s) of each study as described in the original publication. Results represent the key findings directly related to *IGF2* function, growth, metabolism, or meat/milk quality. Research methods include the primary techniques used for gene expression, protein analysis, or histological evaluation. Figure 1 (goat liver cell culture and porcine embryo fibroblast cells) is referenced where applicable to illustrate specific *in vitro* outcomes. References correspond to the numbered list provided in the manuscript. Abbreviations: FCR = feed conversion ratio, GLUT4 = glucose transporter type 4, *IGF2* = insulin-like growth factor 2, MTT = 3-(4,5-dimethylthiazol-2-yl)-2,5-diphenyltetrazolium bromide, PI3K = phosphoinositide 3-kinase, RT-qPCR = reverse transcription quantitative polymerase chain reaction.

*In vivo* studies were conducted in live animals; *in vitro* studies used primary or immortalized cell cultures. Research purposes indicate the main objective(s) of each study as described in the original publication. Results represent the key findings directly related to *IGF2* function, growth, metabolism, or meat/milk quality. Research methods include the primary techniques used for gene expression, protein analysis, or histological evaluation. Figure 1 (goat liver cell culture and porcine embryo fibroblast cells) is referenced where applicable to illustrate specific *in vitro* outcomes. References correspond to the numbered list provided in the manuscript. Abbreviations: FCR = feed conversion ratio, GLUT4 = glucose transporter type 4, *IGF2* = insulin-like growth factor 2, MTT = 3-(4,5-dimethylthiazol-2-yl)-2,5-diphenyltetrazolium bromide, PI3K = phosphoinositide 3-kinase, RT-qPCR = reverse transcription quantitative polymerase chain reaction.

Complementary *in vitro* studies provide mechanistic insights into molecular pathways regulated by *IGF2*. Stimulation by *IGF2* activates the PI3K–AKT–mTOR signaling pathway in chicken muscle cell cultures, accelerating muscle protein synthesis and promoting anabolic activity, as confirmed by Western blot, RT-qPCR, and immunofluorescence analyses [145]. In goat hepatocyte cultures, *IGF2* regulates lipid metabolism by reducing intracellular lipid accumulation, suggesting its involvement in hepatic energy homeostasis [146]. Moreover, *IGF2* enhances myogenic differentiation in duck muscle progenitor cells, increasing myotube formation and reinforcing its role in skeletal muscle development [147]. Similar proliferative and anabolic effects have been observed in bovine myoblast cultures, where *IGF2* stimulation increases cell proliferation and protein synthesis [148]. *IGF2* also upregulates downstream metabolic genes within the PI3K/AKT pathway and increases GLUT4 expression in porcine embryo fibroblast cells, highlighting its role in glucose uptake and metabolic efficiency [149].

Increased *IGF2* expression has been positively linked to greater muscle mass, better feed efficiency, and faster growth rates in pigs, cattle, and poultry [150]. Targeted modulation of *IGF2* expression offers a promising strategy to boost lean meat production without harming animal health or welfare [151]. Regulation of *IGF2* helps improve carcass quality, and higher *IGF2* activity is generally tied to more efficient nutrient use [152]. Mutations and polymorphisms in the *IGF2* gene have been associated with increased body weight and muscle growth in livestock. A well-known example is a mutation within the *IGF2* regulatory element that causes elevated muscle-specific expression, resulting in enhanced muscle development and better carcass traits [153]. Advances in genomic selection technology enable breeders to find animals with favorable *IGF2* variants for use in breeding programs aimed at achieving optimal growth and improved feed efficiency [154]. However, interactions between genetic background and environmental factors, including nutrition, management practices, and overall husbandry conditions, strongly influence how *IGF2* is expressed phenotypically [155]. As a molecular link between genetics and the endocrine system, the *IGF2* gene affects hormonal balance and metabolic efficiency. The *IGF2* gene also plays a role in the insulin signaling pathway that controls glucose uptake, protein synthesis, and energy partitioning, helping to optimize animal performance under varying environmental and nutritional conditions [156]. Incorporating *IGF2* expression analysis into livestock selection programs can aid in predicting superior breeds and enhancing understanding of animal resilience, adaptability, and long-term production potential [157].

## *IGF2* IMPACT ON ANIMAL HEALTH AND WELFARE

Changes in *IGF2* expression can also affect the overall health and welfare of animals [158]. Although *IGF2* plays a central role in regulating muscle development and energy metabolism, dysregulation of its expression may disrupt metabolic homeostasis and increase the risk of adverse health outcomes [159]. Imbalance in *IGF2* signaling can affect coordination between muscle accretion and lipid metabolism, potentially leading to metabolic disorders [160]. The *IGF2* gene can also influence the endocrine system, particularly in interaction with other growth-related hormones such as insulin and *IGF1*. The *IGF2* gene interacts with insulin to regulate glucose utilization in the body, and increased *IGF2* expression may alter blood glucose regulation [161]. Understanding the complex interactions between *IGF2* and other hormones is essential to prevent negative effects on animal health. Changes in *IGF2* expression may also influence animal behavior in addition to physical effects [162]. Alterations in metabolic processes and hormonal balance can affect both mental and physical well-being. Stress caused by rapid muscle growth or disturbances in energy metabolism can reduce quality of life, weaken the immune system, and increase susceptibility to disease [163].

Growth-related hormones, including *IGF2*, influence immune cell function, and changes in *IGF2* levels can impact the body’s ability to respond to infection or stress [164]. Excessive *IGF2* expression may hinder optimal immune responses, increasing vulnerability to infection and disease [165]. While animals might stay healthy after genetic modification, monitoring hormonal balance and its effects on immune function remains essential. Increasing *IGF2* expression benefits meat production efficiency; however, potential side effects, especially those involving hormonal balance, need careful assessment [166]. Investigating hormonal interactions and the long-term effects of *IGF2* genetic modification is crucial to identify potential health risks [167].

Biotechnological modification of *IGF2* expression through genetic engineering has shown significant potential for enhancing livestock productivity [168]. However, maintaining physiological balance is essential for animal welfare. Uncontrolled alterations in *IGF2* expression can disrupt metabolic stability, for example by causing insulin problems or increased blood glucose levels that may lead to insulin resistance [169]. Excessive *IGF2* activity might also worsen metabolic disorders, including obesity-related issues, thereby decreasing productivity and harming animal health [170]. Therefore, strategies involving genetics and biotechnology targeting *IGF2* should be applied within a carefully regulated framework that considers animal health, welfare, and production objectives [171].

## CONCLUSION

The present review highlights the central role of *IGF2* as a key molecular regulator of growth, muscle development, metabolic efficiency, and overall production performance in livestock. Evidence collected from genetic, nutritional, physiological, and biotechnological studies consistently demonstrates that *IGF2* significantly influences economically important traits, including body weight gain, muscle fiber formation, carcass quality, feed conversion efficiency, reproductive performance, and metabolic regulation. Experimental findings from both *in vivo* and *in vitro* studies confirm that modulation of *IGF2* expression affects major signaling pathways such as PI3K–AKT–mTOR, which regulate protein synthesis, cell proliferation, and energy utilization. Polymorphisms within the *IGF2* gene, particularly in regulatory regions, have been strongly associated with improved growth traits in cattle, pigs, poultry, and small ruminants, supporting its application as a reliable candidate gene for MAS and genomic selection programs. In addition, recent advances in biotechnology, including CRISPR–Cas9 genome editing, epigenetic modulation, and transcriptomic approaches, have provided new opportunities to regulate *IGF2* expression to enhance livestock productivity while maintaining desirable carcass characteristics.

From a practical standpoint, combining *IGF2*-based genetic selection with optimized nutritional management and improved husbandry practices presents a promising approach to increasing livestock production efficiency sustainably. Animals with advantageous *IGF2* variants typically show faster growth, better feed efficiency, and enhanced meat quality, which can directly boost farm profitability and industry competitiveness. Additionally, understanding how *IGF2* expression interacts with environmental factors such as diet, stress, and management conditions enables the development of precision breeding and feeding strategies tailored to specific production systems. These methods can lead to more efficient resource use, lower production costs, and higher product quality, supporting the long-term sustainability of the livestock industry.

One of the major strengths of the current review is the integration of information from multiple disciplines, including molecular genetics, animal nutrition, physiology, and biotechnology, providing a comprehensive understanding of *IGF2*-mediated growth regulation across different livestock species. However, several limitations remain. Many studies have been conducted in specific species or under controlled experimental conditions, which may limit the direct application of the results to diverse production environments. In addition, the long-term effects of manipulating *IGF2* expression, particularly through genetic engineering and epigenetic modification, are not yet fully understood and require careful evaluation to ensure animal health, welfare, and consumer acceptance.

Future research should emphasize large-scale multi-species studies to clarify how *IGF2* polymorphisms, nutritional factors, and environmental conditions interact within practical farming systems. Additional research is also necessary to understand the epigenetic regulation of *IGF2*, its interactions with other growth-related genes such as *GH*, *GHR*, and *MSTN*, and its role in immune function and metabolic stability. Developing safe and ethically acceptable biotechnological methods for controlling *IGF2* expression will be crucial for successfully applying this gene in modern breeding programs.

In conclusion, *IGF2* is a key molecular target that connects genetics, nutrition, and physiology in livestock production. Strategic use of *IGF2* through selective breeding, nutritional adjustments, and controlled biotechnological methods has strong potential to enhance growth, improve production efficiency, and boost product quality while safeguarding animal health and welfare. Ongoing research that combines molecular biology with practical livestock management is crucial to fully harness the benefits of *IGF2* for sustainable and economically viable animal production systems.

## AUTHORS’ CONTRIBUTIONS

SRA, SS, MAA, ARK, ML, and EJK: Contributed to the literature search, conceptualization, and initial drafting of the manuscript. ML, MAA, RZA, WW, ZNAR, SRA, and SHW: Critical revision and substantive editing of the manuscript for important intellectual content. MAA, WW, WPL, RZA, and TRF: Manuscript drafting and subsequent revisions. MS, WW, RR, WPL, SRA, RZA, and ARK: Managed reference organization, formatting, and verification. All authors have read and approved the final version of the manuscript and agree to be accountable for all aspects of the work.
